# Nursing Supports for Managing Nausea and Vomiting in Patients with Cancer Having a Prognosis of Months or Weeks: A Multisite Cross-Sectional Study of Palliative Care Nurses in Japan

**DOI:** 10.1089/pmr.2024.0093

**Published:** 2025-02-10

**Authors:** Masamitsu Kobayashi, Kohei Kajiwara, Kimiko Nakano, Yusuke Kanno, Miharu Morikawa, Yoshinobu Matsuda, Jun Kako

**Affiliations:** ^1^ Graduate of Nursing Science, St. Luke’s International University, Tokyo, Japan.; ^2^ Japanese Red Cross Kyushu International College of Nursing, Munakata, Japan.; ^3^ Nursing Department, Mie University Hospital, Tsu, Japan.; ^4^ Graduate School of Health Care Sciences, Institute of Science Tokyo, Tokyo, Japan.; ^5^ Graduate School of Medicine, Kyoto University, Kyoto, Japan.; ^6^ Department of Psychosomatic Internal Medicine, NHO Kinki Chuo Chest Medical Center, Sakai, Japan.; ^7^ Graduate School of Medicine, Mie University, Tsu, Japan.

**Keywords:** cancer, nausea, nursing support, vomiting

## Abstract

**Purpose:**

This study aimed to clarify the types of nursing support provided by palliative care unit (PCU) nurses in Japan to manage nausea and vomiting in patients with cancer who have a prognosis of months or weeks.

**Methods:**

This multisite cross-sectional study surveyed registered nurses from all 389 PCUs across Japan. Eligible participants were nurses providing direct care to patients. Data were collected via online surveys from October 2023 to March 2024. The frequency of 13 types of nursing supports for nausea and vomiting was evaluated using a five-point Likert scale, stratified by patient prognosis (months or weeks).

**Results:**

Of the 389 PCUs invited, 162 (41.6%) consented to participate. A total of 2448 nurses were invited, of which 539 (22.3%) responded. The most frequently implemented nursing supports were “avoiding unpleasant odors,” “providing shaved ice or ice chips,” “providing fresh air,” and “gargling with cold water.” These were consistently practiced by many nurses, regardless of patient prognosis. Conversely, specialized supports such as “reiki,” “acupressure,” “guided relaxation exercises,” “therapeutic touch,” and “foot reflexology” were rarely or seldom used.

**Conclusion:**

Noninvasive, simple nursing supports that do not require specialized knowledge or skills were frequently provided to patients with cancer who were experiencing nausea and vomiting, irrespective of their prognosis. However, nursing supports that require specialized knowledge and skills were rarely used. Further research is needed to evaluate the effectiveness of these nursing supports.

## Introduction 

Nausea and vomiting are prevalent symptoms among patients with advanced cancer and can arise from various factors, including medications such as chemotherapy and opioids, gastrointestinal motility abnormalities, and central nervous system or psychological factors.^1,2^ These distressing symptoms are often multifactorial,^3^ can be directly or indirectly associated with the disease, and significantly impact both the physical and mental health of patients.^4^ In patients with terminal cancer, managing nausea and vomiting can be particularly challenging, as identifying and addressing the underlying causes is often difficult or impossible. Therefore, patient care primarily focuses on symptom relief. Antiemetic medications are typically recommended as the primary intervention for nausea and vomiting in patients with cancer.^5–7^ However, integrating nonpharmacological approaches is also considered beneficial in alleviating patient discomfort.^4,8^

Nurses are integral to providing nonpharmacological care for patients with terminal cancer. However, evidence supporting nursing practices for nausea and vomiting remains insufficient, and in clinical settings, nurses may rely on personal experience. This lack of evidence-based support has led to a limited understanding of the specific care practices nurses implement and the extent of their application. Therefore, this study aimed to clarify the state of nursing support provided by palliative care nurses in Japan for managing nausea and vomiting in patients with cancer who have a prognosis of months or weeks. The findings from this study are expected to provide foundational insights into current practices in clinical settings and to contribute to improving the quality of nursing support in the future.

## Materials and Methods

This study is an ancillary analysis of a large-scale survey conducted among nurses working in palliative care units (PCUs) across Japan, with prior findings addressing dyspnea and caregiving burden.^9,10^

### Study design and setting

This multisite, cross-sectional study involved nurses from PCUs across Japan. In Japan, specialized palliative care services include both PCUs and palliative care teams.^11^ The survey targeted all 389 PCUs nationwide, where admission charges for PCUs had been accepted as of September 14, 2023.

### Participants

The study included nurses providing direct patient care in PCUs. The exclusion criteria were as follows: (1) managers of a PCU (e.g., head nurse), (2) nurses from other wards who were temporarily assigned to PCUs for training, and (3) nurses from other hospitals who were temporarily assigned to PCUs.

All target facilities were invited to participate, and explanatory documents were sent to those that agreed to participate. Nurses from consenting facilities then completed an online questionnaire, with data collected from October 1, 2023, to March 31, 2024, via LimeSurvey Cloud provided by LimeSurvey GmbH (https://www.limesurvey.org/ja).

### Measurements

The online survey assessed nursing supports related to managing nausea and vomiting in patients with cancer who had a prognosis of months or weeks. Nursing supports were identified based on findings from a scoping review on nursing support for nausea and vomiting in patients with cancer and a Delphi study.^12,13^ In addition, input from a preliminary survey of nine experienced PCU nurses helped refine the list of identified nursing supports. The final set included 13 types of nursing supports: foot reflexology, acupressure, reiki, guided relaxation exercises, aromatherapy, abdominal massage, therapeutic touch, psychosocial support, self-symptom monitoring, gargling with cold water, providing fresh air, providing shaved ice or ice chips, and avoiding unpleasant odors. These supports were rated on a five-point Likert scale (1: rarely; 2: seldom; 3: sometimes; 4: frequently; 5: very frequently), with nurses indicating the extent of support usage based on whether the patient’s prognosis was of months or weeks. Participant demographic information, including sex, age, years of nursing and PCU experience, educational background, and qualifications, was also collected.

### Statistical analysis

Descriptive analyses were conducted to analyze demographic information and the frequency of nursing supports. Statistical analysis was performed using EZR, a component of the R software (Saitama Medical Center, Jichi Medical University, Saitama, Japan).^14^

### Research ethics

This study was approved by the Clinical Research Ethics Review Committee of Mie University Hospital (U2023-011) and registered with the University Hospital Medical Information Network, Japan (UMIN0000 52329).

## Results

All 389 PCUs in Japan were invited to participate in the study, with 162 facilities (41.6%) consenting to participate. A total of 2448 nurses from these 162 PCUs were invited to complete the questionnaire, and 539 (22.3%) responded. The average age of the respondents was 42.3 ± 9.8 years, with an average of 18.6 ± 9.6 years of nursing experience and 4.8 ± 4.3 years of experience specifically in PCU settings (Table 1).

**Table 1.** Participant Characteristics (*n* = 539)

	*n* (%)	
Sex		
Female	511	(94.8)
Male	26	(4.8)
Prefer not to answer	2	(0.4)
Educational background		
Nursing vocational school (including junior college)	456	(84.6)
Nursing university	64	(11.9)
Master’s program (predoctoral program)	10	(1.9)
Doctoral program (postdoctoral program)	1	(0.2)

	Mean (SD^a^)	
Age (years)	42.3	(9.8)
Years of nursing experience	18.6	(9.6)
Years of experience in palliative care unit	4.8	(4.3)

^a^ SD: standard deviation.

Among patients with a prognosis of months, the most frequently (frequently/very frequently) reported nursing supports included “avoiding unpleasant odors,” followed by “providing shaved ice or ice chips,” “providing fresh air,” and “gargling with cold water,” and those that were rarely or seldom utilized were “reiki,” “acupressure,” “guided relaxation exercises,” “therapeutic touch,” and “foot reflexology.” These patterns remained consistent for patients with a prognosis of weeks as well (Table 2).

**Table 2.** Nursing Supports for Nausea and Vomiting Management in Patients with Cancer, Stratified by Prognosis (*n* = 539)

	Prognosis of months	Prognosis of weeks
Nursing support	*n* (%)	*n* (%)
Foot reflexology		
Rarely	460 (85.3)	462 (85.7)
Seldom	46 (8.5)	42 (7.8)
Sometimes	17 (3.2)	16 (3.0)
Frequently	12 (2.2)	16 (3.0)
Very frequently	4 (0.7)	3 (0.6)
Acupressure		
Rarely	479 (88.9)	478 (88.7)
Seldom	36 (6.7)	38 (7.1)
Sometimes	15 (2.8)	15 (2.8)
Frequently	7 (1.3)	6 (1.1)
Very frequently	2 (0.4)	2 (0.4)
Reiki		
Rarely	511 (94.8)	512 (95.0)
Seldom	13 (2.4)	14 (2.6)
Sometimes	9 (1.7)	7 (1.3)
Frequently	4 (0.7)	5 (0.9)
Very frequently	2 (0.4)	1 (0.2)
Guided relaxation exercises		
Rarely	461 (85.5)	465 (86.3)
Seldom	53 (9.8)	48 (8.9)
Sometimes	18 (3.3)	20 (3.7)
Frequently	5 (0.9)	3 (0.6)
Very frequently	2 (0.4)	3 (0.6)
Aromatherapy		
Rarely	390 (72.4)	389 (72.3)
Seldom	74 (13.7)	75 (13.9)
Sometimes	64 (11.9)	61 (11.3)
Frequently	7 (1.3)	7 (1.3)
Very frequently	4 (0.7)	6 (1.1)
Abdominal massage		
Rarely	275 (51.0)	279 (51.8)
Seldom	134 (24.9)	127 (23.6)
Sometimes	105 (19.5)	106 (19.7)
Frequently	19 (3.5)	21 (3.9)
Very frequently	6 (1.1)	6 (1.1)
Therapeutic touch		
Rarely	476 (88.3)	475 (88.1)
Seldom	30 (5.6)	28 (5.2)
Sometimes	23 (4.3)	21 (3.9)
Frequently	7 (1.3)	10 (1.9)
Very frequently	3 (0.6)	5 (0.9)
Psychosocial support		
Rarely	131 (24.3)	138 (25.7)
Seldom	115 (21.4)	123 (22.9)
Sometimes	188 (34.9)	173 (32.2)
Frequently	77 (14.3)	76 (14.1)
Very frequently	27 (5.0)	28 (5.2)
Self-symptom monitoring		
Rarely	361 (67.0)	379 (70.3)
Seldom	100 (18.6)	104 (19.3)
Sometimes	53 (9.8)	32 (5.9)
Frequently	18 (3.3)	17 (3.2)
Very frequently	7 (1.3)	7 (1.3)
Gargling with cold water		
Rarely	46 (8.5)	42 (7.8)
Seldom	39 (7.2)	62 (11.5)
Sometimes	230 (42.7)	230 (42.7)
Frequently	164 (30.4)	153 (28.4)
Very frequently	60 (11.1)	52 (9.6)
Providing fresh air		
Rarely	20 (3.7)	14 (2.6)
Seldom	36 (6.7)	40 (7.4)
Sometimes	189 (35.1)	192 (35.6)
Frequently	202 (37.5)	202 (37.5)
Very frequently	92 (17.1)	91 (16.9)
Providing shaved ice or ice chips		
Rarely	27 (5.0)	23 (4.3)
Seldom	33 (6.1)	39 (7.2)
Sometimes	150 (27.8)	142 (26.3)
Frequently	220 (40.8)	219 (40.6)
Very frequently	109 (20.2)	116 (21.5)
Avoiding unpleasant odors		
Rarely	14 (2.6)	13 (2.4)
Seldom	18 (3.3)	17 (3.2)
Sometimes	135 (25.0)	135 (25.0)
Frequently	253 (46.9)	253 (46.9)
Very frequently	119 (22.1)	121 (22.4)

## Discussion

This study investigated the nursing support provided by PCU nurses in Japan for managing nausea and vomiting in patients with cancer who had a prognosis of months or weeks. The results showed that the frequency of nursing support was nearly the same, regardless of the patient’s prognosis.

Nursing supports such as “avoiding unpleasant odors,” “providing shaved ice or ice chips,” “providing fresh air,” and “gargling with cold water” were frequently implemented in both prognosis groups. These supports are clinically favorable because they are noninvasive, low-cost, and easily accessible. In addition, patients can easily self-administer these supports at their own pace, with family assistance where needed. Among these supports, avoiding unpleasant odors is particularly effective for managing nausea and vomiting, as strong or offensive smells can activate the vomit center in the brain, primarily through the olfactory system.^15^ Therefore, maintaining a clean personal environment and avoiding any odors that may cause discomfort are crucial in managing nausea and vomiting in patients with cancer. Providing shaved ice or ice chips may alleviate nausea and vomiting by cooling the oral cavity; in patients undergoing chemotherapy, oral cryotherapy has been shown to reduce oral discomfort and associated nausea and vomiting.^16^ However, although this approach provides comfort and potentially reduces these symptoms, its benefits in patients with terminal cancer remain uncertain. Providing fresh air improves patient comfort and is a common practice in palliative care settings and for patients undergoing cancer treatment,^17^ although the mechanism by which it reduces nausea and vomiting is not understood. Gargling with cold water may relieve nausea by cooling the mouth and throat and alleviating xerostomia and bad taste, both of which can contribute to nausea and vomiting. Although drinking cold water has been shown to reduce chemotherapy-induced nausea and vomiting,^18^ it may not be feasible in terminal care settings, making assisted gargling a practical alternative.

In contrast, nursing supports such as “reiki,” “acupressure,” “guided relaxation exercises,” “therapeutic touch,” and “foot reflexology” were rarely provided in either prognosis period. The infrequent use of these supports may be attributed to the specialized knowledge and skills required for their implementation. In addition, limited evidence supports their effectiveness, highlighting the need for further research.^12^ If future studies confirm their effectiveness, it will be necessary to create an environment that facilitates their adoption.

### Limitations

This study had several limitations. First, the low response rate might have limited the representativeness of the findings, thereby restricting the generalizability of our results. Second, as the participants in this study were nurses working exclusively in Japan, the findings might have been influenced by cultural factors unique to the Japanese health care setting. Therefore, the broader applicability of these results to other countries may be limited. Third, the reported frequencies of nursing supports may not accurately reflect their actual implementation in clinical settings. Despite these limitations, we believe this study provides valuable insights that can inform future research and clinical practice.

## Conclusions

This study found that Japanese PCU nurses provide similar support for managing nausea and vomiting in patients with terminal cancer, regardless of prognosis. The most frequently implemented nursing supports were “avoiding unpleasant odors,” “providing shaved ice or ice chips,” “providing fresh air,” and “gargling with cold water.” Future research should evaluate the effectiveness of these frequently implemented nursing supports to optimize care for patients with terminal cancer.
